# Gut microbiome features co‐occur in different groups of Alzheimer’s disease phenotypes and genotypes

**DOI:** 10.1002/alz.090763

**Published:** 2025-01-09

**Authors:** Jea Woo Kang, Lora A. Khatib, Amanda H. Dilmore, Margo B. Heston, Tyler K. Ulland, Sterling C. Johnson, Sanjay Asthana, Cynthia M. Carlsson, Nathaniel A. Chin, Rob Knight, Rima Kaddurah‐Daouk, Federico E. Rey, Barbara B. Bendlin, Alzheimer's Gut Microbiome Project Consortium (AGMP)

**Affiliations:** ^1^ Wisconsin Alzheimer's Disease Research Center, University of Wisconsin School of Medicine and Public Health, Madison, WI USA; ^2^ University of California, San Diego, La Jolla, CA USA; ^3^ Department of Pathology and Laboratory Medicine, University of Wisconsin‐Madison, Madison, WI USA; ^4^ Wisconsin Alzheimer's Disease Research Center, Madison, WI USA; ^5^ Department of Psychiatry and Behavioral Sciences, Duke University, Durham, NC USA; ^6^ Department of Bacteriology, University of Wisconsin‐Madison, Madison, WI USA; ^7^ Duke University, Durham, NC USA

## Abstract

**Background:**

Gut microbiome features are known to be different in Alzheimer’s disease (AD) patients compared with cognitively normal. Evidence suggests that the gut microbiota may be related to the pathology of AD. We examined the association between the gut microbiome and AD phenotypes and genotypes.

**Method:**

Cross‐sectional analysis was performed on the filtered microbiome count table including 246 participants from the Microbiome in AD Risk Study. The filtered taxonomic count table was used to analyze differential abundance using the BIRDMAn pipeline. To determine the differences in the microbiota features derived from each cognitive, amyloid, and ApoE4 group, we implemented statistical models that had microbiome features as outcome variables and each cognitive, amyloid, and ApoE4 group as predictor variable adjusting for covariates including age, sex, BMI, Bristol type, and age difference between fecal collection and measurements of each predictor variable.

**Result:**

Each cognitive, amyloid, and ApoE4 group showed differentially abundant microbiota features. Top microbial features (more abundant in AD, amyloid +, ApoE4 +) that co‐occurred with all three groups were mostly Bacteroides spp., Collinsella spp., and Fusobacterium spp. Bottom microbial features (less abundant in AD, amyloid +, ApoE4 +) that co‐occurred with all three groups were mostly Dialister spp. and Prevotella spp.

**Conclusion:**

The same microbial species were more abundant and less abundant in clinical, preclinical, and genetic risk groups. These results suggest candidate microbes that can be stratified in analyses that involve associations between gut microbiome and AD pathology.

